# Two Decades of Change in Childbirth Care in Cambodia (2000–2021): Disparities in Ceasarean Section Utilization Between Public and Private Facilities

**DOI:** 10.1186/s41256-025-00429-7

**Published:** 2025-07-31

**Authors:** Yanqin Zhang, Dyna Khuon, Vonthanak Saphonn, Peng Jia, Qian Long

**Affiliations:** 1 Global Health Research Center, Duke Kunshan University, No. 8 Duke Avenue, Kunshan, 215316 Jiangsu China; 2 Department of Public Health, University of Health Sciences, Phnom Penh, Cambodia; 3 School of Resource and Environmental Sciences, Wuhan University, Wuhan, China

**Keywords:** Caesarean section, Health policy, Health equity, Health system, Public and private healthcare

## Abstract

**Background:**

Cambodia, a lower-middle-income country, confronts challenges related to childbirth safety. This study investigated the utilization of childbirth care across public and private health facilities, with a particular focus on the use of caesarean section (C-section). It also examined disparities in service utilization across urban and rural areas and among different socioeconomic statuses.

**Methods:**

This study used cross-sectional data from the Demographic and Health Surveys conducted in Cambodia in 2000, 2005, 2010, 2014 and 2021–22. Descriptive analyses were performed to elucidate changes in place of delivery and C-section rates by public and private health facilities. Logistic regressions were applied using data from 2010 to 2021 to identify factors associated with C-section.

**Results:**

The facility-based delivery rate significantly increased to 96.5% by 2021, while the overall C-section rate rose to 15.5%. Within public facilities, the C-section rate grew from 5.1% in 2010 to 9.7% in 2021, consistently higher in urban areas compared to rural ones. Notably, C-section utilization in public facilities did not significantly vary among different wealth index groups. From 2010 to 2021, the C-section rates in private facilities surged from 11.0% to 48.1%, with urban and rural rates reaching 50.5% and 45.7%, respectively. In 2021, the richest and richer groups accounted for most C-section deliveries in private facilities, constituting 38.5% and 28.8%, respectively. C-section use was significantly higher in 2021 compared to 2010 (Adjusted OR 3.32, 95% CI [2.72, 4.07]). Women over 20 years old, living in Central Plain, from richer or richest households, had secondary and higher education level, with female household head and had only one child were more likely to undergo a C-section than other women.

**Conclusions:**

The private facilities have significantly driven the increase in C-sections, particularly among wealthier economic groups. Strengthening health system governance and promoting public–private partnerships are vital to curb C-section overuse and ensure equitable and effective childbirth care coverage.

## Introduction

Caesarean section (C-section) is a critical life-saving intervention for managing childbirth complications [1, 2]. However, over the past three decades, global C-section rates have increased substantially due to non-medical factors such as maternal requests and financial incentives, especially in private health settings [3–5]. Such unnecessary C-sections increase maternal and neonatal morbidity and mortality risks, including surgical infections and potential long-term fertility complications [3, 6–8]. In many low- and lower-middle-income countries, simultaneous underuse and overuse of C-sections reflect systemic inequities, uneven resource allocation, and variations in healthcare quality [6, 9, 10]. Recognizing these issues, the World Health Organization (WHO) recommended in 2015 that C-sections should be limited to medical necessity, noting evidence that rates above 10% do not further reduce maternal and neonatal mortality [11].

Cambodia, a lower-middle-income country in Southeast Asia, significantly reduced its maternal mortality ratio (MMR) from 606 per 100,000 live births in 2000 to 218 per 100,000 live births in 2020 [12]. Maternity care in Cambodia is provided through a mixed public–private delivery system [13]. In the public sector, primary health centers offer basic childbirth services, while C-sections are referred to higher-level hospitals [14, 15]. In 1994, the government launched the National Reproductive Health Program to promote facility-based deliveries to ensure childbirth safety [16]. This was followed by the introduction of the Emergency Obstetric and Newborn Care (EmONC) Improvement Plan, focusing on strengthening the capacity of public health facilities to provide emergency care and setting a national caesarean delivery target of 10% by 2020 [17, 18].

Although prior studies in Cambodia have largely examined maternity care coverage from a decade ago, there is limited research investigating recent utilization trends, specifically the contribution of the private health sector to childbirth care. This study examined access to childbirth services, including C-section utilization, across public and private health sectors, analyzing associated urban–rural and socioeconomic disparities to inform strategies for promoting equitable and high-quality childbirth care.

## Methods

### Data sources and population

This study used cross-sectional data from Cambodia Demographic and Health Surveys (CDHS) in 2000, 2005, 2010, 2014 and 2021–22 (later refer to 2021), conducted by the National Institute of Statistics and the Ministry of Health [19–23]. The CDHS employed a two-stage sampling design, first selecting clusters from enumeration areas defined in the General Population Census [24], and then randomly sampled households within these clusters to provide representative estimates at national and regional levels [19–23]. All women aged 15–49, whether permanent residents or overnight visitors of the selected households, were eligible for interview. More detailed sample designs can be found elsewhere [19–23].

This study used women datasets of the Cambodia surveys, including women who had a live birth in the past three years prior to the surveys. If a participant reported multiple live births within this period, only data on the most recent birth were used to avoid overrepresentation of high-parity women.

### Outcome measures

The outcome measures included the place of delivery and C-section rate. The surveys asked women ‘Where did you give birth to (child name)?’. This question was labelled as ‘place of delivery’ with responses of home, public sector (including public hospital, health center/post) and private sector (private hospital, private clinic and private cabinet). The private sector primarily represented for-profit facilities, as the usage of private non-for-profit facilities was minimal. In the surveys, women were also asked ‘Was (child name) delivered by caesarean?’ with a binary response of yes or no. We calculated the C-section rate by dividing the number of caesarean deliveries by the total number of births.

### Demographic and socioeconomic factors

Demographic and socioeconomic variables have been recoded to residence (urban and rural), region (Central Plain, Tonle Sap, Coastal and Sea, and Plateau and Mountains), maternal age (≤ 19, 20–29 and 30 +), religion (Buddhist and other), education (no education, incomplete primary, primary, secondary and higher), health insurance coverage (yes and no), type of health insurance (social and private), wealth index (poorest, poorer, middle, richer and richest), partner’s education (no education, incomplete primary, primary, secondary and higher), sex of household head (male and female), decision-making on respondent’s health care (independently, jointly and not involve) and decision-making on household (independently, not entirely independent, jointly and not involve). The DHS team computes the wealth index by assessing household assets, construction materials, and water and sanitation facilities to reflect each household's overall living standard [25].

### Maternal history and child-related factors

Maternal history and child-related factors included the number of antenatal visits (0, 1–3, 4–7 and 8 +) during this pregnancy, parity (total number of children ever born), size of child at birth (average, larger than average, smaller than average), twins or multiple births (yes and no) and sex of child (male and female). According to the DHS survey design, the size of child at birth was reported subjectively by the respondents.

### Statistical analysis

Descriptive analysis was conducted to illustrate the demographic and socioeconomic characteristics of the study sample and to observe the changes in the place of delivery and C-section usage from 2000 to 2021. The distributions of place of delivery and C-section usage were further examined across public and private health facilities. Additionally, these distributions were stratified by residence settings and household wealth index. The Chi-Square test was used to test the difference over the study period.

Most women gave birth at home before 2010, thus we only included year 2010, 2014 and 2021 into the regression analysis to identify factors associated with C-section usage. The Logistic regression model initially included all potential predictors. The stepwise regression was then employed to identify the most significant predictors of C-section usage. It began with a full model that included all potential predictors and iteratively removed the least significant variables. The selection process was guided by the Akaike Information Criterion [23] and continued until no further enhancements were observed. The Hosmer–Lemeshow Test was used to assess the goodness of fit for the regression model by comparing the observed and predicted probabilities across subgroups within the dataset [26]. All analyses were conducted using R version 4.3.3 [27].

## Results

### Participants

The study included 21,984 women who had a live birth within three years prior to the surveys conducted between 2000 and 2021 (Table 1). Urban residency among women increased from 13.9% in 2000 to 32.4% in 2021. Regionally, there was a decline in Tonle Sap in the same period, decreasing from 37.1% to 29.7%.

**Table 1:** Background characteristics of women who had a live birth in the past three years prior to the DHS surveys, Cambodia, 2000 –2021

	2000 (N = 4396) n (%)	2005 (N = 4495) n (%)	2010 (N = 4561) n (%)	2014 (N = 4077) n (%)	2021–22 (N = 4455) n (%)	*P* value
Demographic and socio-economic						
*Residence*^*1*^						
Urban	613 (13.9%)	934 (20.8%)	1165 (25.5%)	1132 (27.8%)	1445 (32.4%)	< 0.001
Rural	3772 (85.8%)	3505 (78.0%)	3341 (73.3%)	2896 (71.0%)	2980 (66.9%)	
*Region*^*2*^						
Central Plain^3^	1297 (29.5%)	1169 (26.0%)	1289 (28.3%)	1164 (28.6%)	1287 (28.9%)	< 0.001
Tonle Sap^4^	1631 (37.1%)	1657 (36.9%)	1646 (36.1%)	1413 (34.7%)	1324 (29.7%)	
Coastal and Sea^5^	505 (11.5%)	438 (9.7%)	433 (9.5%)	388 (9.5%)	604 (13.6%)	
Plateau and Mountains^6^	952 (21.7%)	1175 (26.1%)	1138 (25.0%)	1063 (26.1%)	1210 (27.2%)	
*Maternal age*						
Mean (SD)	29.3 (6.8)	28.0 (6.8)	27.2 (6.2)	26.7 (5.9)	28.0 (6.2)	
19 and less	339 (7.7%)	353 (7.9%)	351 (7.7%)	373 (9.1%)	362 (8.1%)	< 0.001
20**–**29	1986 (45.2%)	2394 (53.3%)	2873 (62.0%)	2501 (61.3%)	2335 (52.4%)	
30+	2071 (47.1%)	1748 (38.9%)	1337 (29.3%)	1203 (29.5%)	1758 (39.5%)	
*Religion*^7^						
Buddhist	4026 (91.6%)	4146 (92.2%)	4254 (93.3%)	3843 (94.3%)	4281 (96.1%)	< 0.001
Other^8^	367 (8.3%)	347 (7.7%)	306 (6.7%)	232 (5.7%)	174 (3.9%)	
*Education*						
No education	1625 (37.0%)	1243 (27.7%)	921 (20.2%)	527 (12.9%)	555 (12.5%)	< 0.001
Incomplete primary	2042 (46.5%)	2293 (51.0%)	2005 (44.0%)	1535 (37.7%)	1347 (30.2%)	
Primary^9^	691 (15.7%)	878 (19.5%)	1413 (31.0%)	1648 (40.4%)	1965 (44.1%)	
Secondary and higher^10^	38 (0.9%)	81 (1.8%)	222 (4.9%)	367 (9.0%)	588 (13.2%)	
*Health insurance type*						
No insurance	–	–	3761 (82.5%)	3456 (84.8%)	3505 (78.7%)	< 0.001
Social^11^	–	–	718 (15.7%)	534 (13.1%)	731 (16.4%)	
Private	–	–	74 (1.6%)	88 (2.2%)	221 (5.0%)	
*Wealth*^*12*^						
Poorest	–	1275 (28.4%)	1154 (25.3%)	926 (22.7%)	1323 (29.7%)	< 0.001
Poorer	–	1030 (22.9%)	880 (19.3%)	746 (18.3%)	860 (19.3%)	
Middle	–	821 (18.3%)	767 (16.8%)	647 (15.9%)	782 (17.6%)	
Richer	–	696 (15.5%)	800 (17.5%)	732 (18.0%)	886 (19.9%)	
Richest	–	673 (15.0%)	960 (21.0%)	1026 (25.2%)	604 (13.6%)	
*Partner’s education*^**13**^						
No education	953 (21.7%)	762 (17.0%)	579 (12.7%)	385 (9.4%)	440 (9.9%)	< 0.001
Incomplete primary	1903 (43.3%)	1917 (42.6%)	1637 (35.9%)	1344 (33.0%)	1241 (27.9%)	
Primary	1465 (33.3%)	1433 (31.9%)	1744 (38.2%)	1618 (39.7%)	1769 (39.7%)	
Secondary and higher	30 (0.7%)	269 (6.0%)	539 (11.8%)	712 (17.5%)	751 (16.9%)	
*Sex of household head*						
Male	3656 (83.1%)	3823 (85.1%)	3643 (79.9%)	3146 (77.2%)	3296 (74.0%)	< 0.001
Female	740 (16.8%)	672 (14.9%)	918 (20.1%)	931 (22.8%)	1159 (26.0%)	
*Decision-making on own health care*^14^						
Independently	–	–	1713 (37.6%)	1598 (39.2%)	1661 (37.3%)	0.08
Jointly^15^	–	–	2259 (49.5%)	1952 (47.9%)	2198 (49.3%)	
Not involve	–	–	407 (8.9%)	340 (8.3%)	410 (9.2%)	
*Decision-making on household*^16^						
Independently	–	–	297 (6.5%)	277 (5.6%)	244 (5.5%)	0.2
Not entirely independent^17^	–	–	2189 (48.0%)	2218 (54.4%)	2255 (50.6%)	
Jointly	–	–	1809 (39.7%)	1386 (34.0%)	1602 (36.0%)	
Not involve	–	–	55 (1.2%)	40 (1.0%)	150 (3.4%)	
Maternal and child related						
*Number of antenatal visits*^18^						
0	2481 (56.4%)	1309 (29.1%)	533 (11.7%)	191 (4.7%)	86 (1.9%)	
1–3	1508 (34.3%)	2029 (45.1%)	1333 (29.2%)	846 (20.8%)	715 (16.0%)	
4–7	310 (7.1%)	1050 (23.4%)	2252 (49.4%)	2325 (57.0%)	2676 (60.1%)	
8+	21 (0.5%)	87 (1.9%)	429 (9.4%)	703 (17.2%)	973 (21.8%)	
*Parity*						
1	757 (17.2%)	1140 (25.4%)	1492 (32.7%)	1602 (39.3%)	1430 (32.1%)	< 0.001
2	775 (17.6%)	1032 (23.0%)	1242 (27.2%)	1236 (30.3%)	1618 (36.3%)	
3+	2864 (65.2%)	2323 (51.7%)	1827 (40.1%)	1239 (30.4%)	1407 (31.6%)	
*Size of child at birth*^19^						
Average	2386 (54.3%)	1988 (44.2%)	2034 (44.6%)	2186 (53.6%)	2143 (48.1%)	
Larger than average	1228 (27.9%)	1798 (40.0%)	1888 (41.4%)	1428 (35.0%)	1962 (44.0%)	
Smaller than average	637 (14.5%)	676 (15.0%)	501 (11.0%)	442 (10.8%)	332 (7.5%)	
*Twins or multiple birth*						
Yes	56 (1.3%)	43 (1.0%)	39 (0.9%)	38 (0.9%)	25 (0.6%)	
No	4340 (98.7%)	4452 (99.0%)	4522 (99.1%)	4039 (99.1%)	4430 (99.4%)	
*Sex of child*						
Male	2234 (50.8%)	2285 (50.8%)	2347 (51.5%)	2059 (50.5%)	2292 (51.4%)	
Female	2162 (49.2%)	2210 (49.2%)	2214 (48.5%)	2018 (49.5%)	2163 (48.6%)	

^1^11 women in 2000, 56 in 2005, 55 in 2010, 49 in 2014 and 30 in 2021 had missing value

^2^11 women in 2000, 56 in 2005, 55 in 2010, 49 in 2014 and 30 in 2021 had missing value

^3^Central Plain includes Kampong Cham, Tbong Khmum, Kandal, Phnom Penh, Prey Veng, Svay Rieng, and Takeo

^4^Tonle Sap includes Banteay Meanchey, Battambang, Kampong Chhnang, Kampong Thom, Pursat, Siem Reap, Otdar Meanchey, and Pailin

^5^Coastal and Sea includes Kampot, Koh Kong, Preah Sihanouk, and Kep

^6^Plateau and Mountains includes Kampong Speu, Kratie, Mondul Kiri, Preah Vihear, Ratanak Kiri, and Stung Treng

^7^Three women in 2000, two in 2005, one in 2010 and two in 2014 had missing value

^8^Other religion includes Moslem, Christian, other religion and no religion

^9^Primary includes complete primary and incomplete secondary

^10^Secondary and higher includes complete secondary and higher

^11^Social health insurance includes National Social Security Fund and Health Equity Fund

^12^Year 2000 didn’t have any data on wealth index

^13^45 women’s partner in 2000, 114 in 2005, 62 in 2010, 18 in 2014 and 254 in 2021 had missing value

^14^182 women in 2010, 187 in 2014 and 186 in 2021 had missing value

^15^Jointly includes respondent and husband/partner and respondent and other person

^16^Decision-making on household includes making large household purchases, visits to family or relatives and what to do with husband earns; 211 women in 2010, 206 in 2014 and 204 in 2021 had missing value

^17^Women can independently make decision on at least one of household affairs

^18^76 women in 2000, 20 in 2005, 14 in 2010, 12 in 2014 and 5 in 2021 had missing value

^19^145 women in 2000, 33 in 2005, 138 in 2010, 21 in 2014 and 18 in 2021 had missing value

The proportion of women aged over 30 decreased from 47.1% in 2000 to 29.3% in 2010 and subsequently rose to 39.5% by 2021. Educational attainment among women and their partners improved over time, with more than half achieving at least primary education by 2021. Surveys on health insurance coverage began in 2010, indicating persistently low coverage, with only approximately 20% of women holding either social or private insurance by 2021. Between 2005 and 2021, proportions of women in the poorest, poorer, and middle wealth index groups experienced a pattern of initial decline followed by a rise. Notably, the poorest group was 29.7% in 2021, slightly higher than its 2005 level of 28.4%. Additionally, female headship in households rose from 16.8% in 2000 to 26.0% in 2021. About half of the women were involved in joint decision-making regarding their own healthcare and household affairs (Table 1).

The percentage of women who attended at least four antenatal visits had a substantial increase, from 7.6% in 2000 to 81.9% in 2021. Parity trends indicated a significant decrease in families with three or more children during the same period, from 65.2% to 31.6%, leading to a more balanced parity distribution. The gender distribution of children remained approximately equal, with nearly all being single births. Additionally, the proportion of children whose size at birth was larger than average rose from 27.9% in 2000 to 44.0% in 2021 (Table 1).

### Place of delivery

**Fig. 1:**
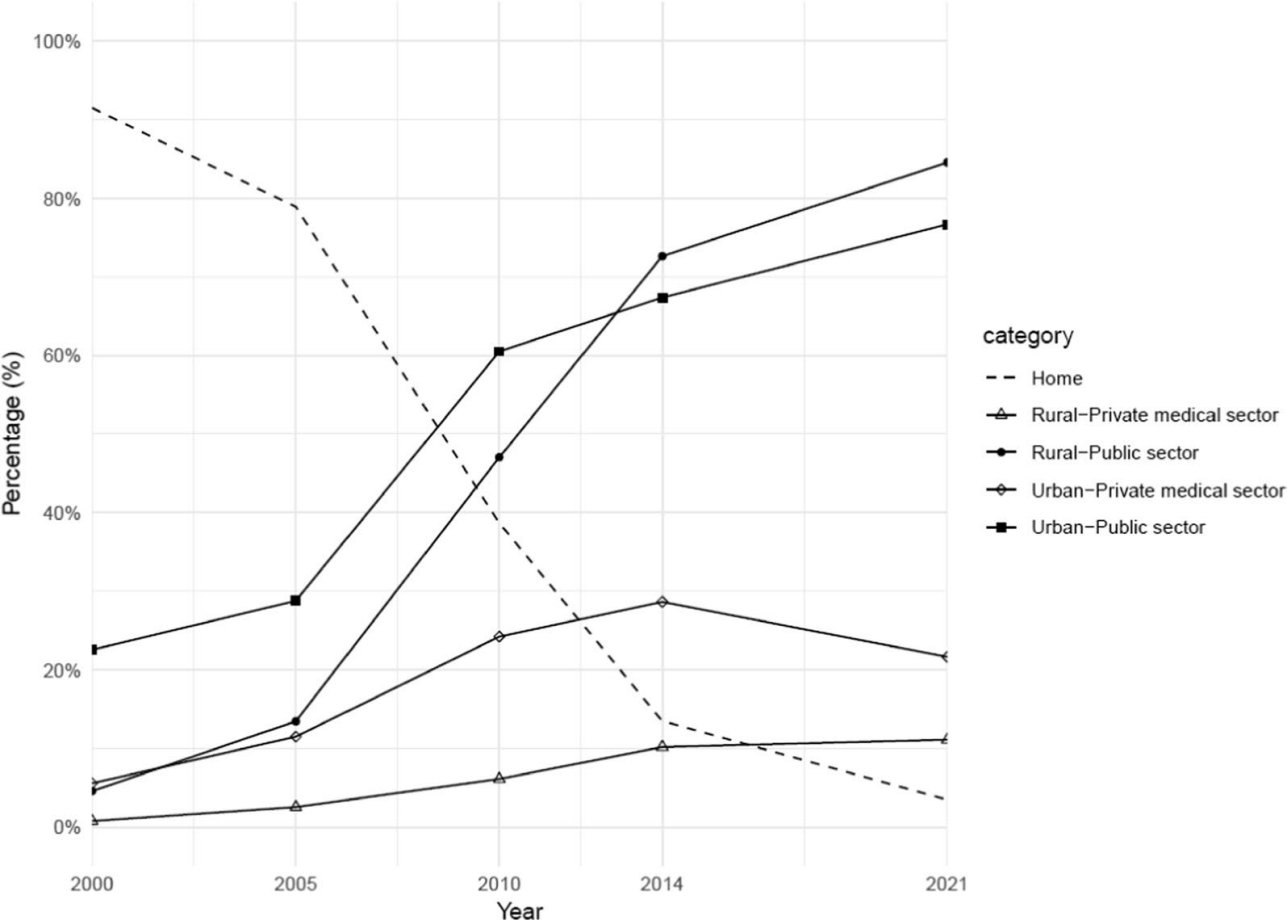
Place of delivery among women who had a live birth in the past three years prior to the DHS surveys, 2000–2021

During the study period, there was a sustained decline in the rate of home delivery, which fell from 91.5% in 2000 to 3.5% in 2021. Concurrently, facility-based deliveries in public health facilities rose markedly from 7.1% to 82.0% over the same timeframe. From 2014 to 2021, the proportion of deliveries in public facilities was higher in rural areas than in urban areas (Fig. 1). Within public health facilities, the distribution of deliveries shifted from being predominantly among the richest group (39.2% in 2005) to a majority from the poorest group (31.9% in 2021) (Table 2).

**Table 2:** Place of delivery by wealth index, 2005–2021

	Place of delivery							
	2005		2010		2014		2021–22	
	Public (N = 755) n (%)	Private (N = 199) n (%)	Public (N = 2293) n (%)	Private (N = 489) n (%)	Public (N = 2896) n (%)	Private (N = 626) n (%)	Public (N = 3626) n (%)	Private (N = 643) n (%)
*Wealth*^1^								
Poorest	87 (11.5%)	2 (1.0%)	429 (18.7%)	19 (3.9%)	612 (21.1%)	26 (4.2%)	1155 (31.9%)	41 (6.4%)
Poorer	101 (13.4%)	7 (3.5%)	393 (17.1%)	30 (6.1%)	561 (19.4%)	49 (7.8%)	750 (20.7%)	86 (13.4%)
Middle	111 (14.7%)	15 (7.5%)	411 (17.9%)	52 (10.6%)	519 (17.9%)	65 (10.4%)	668 (18.4%)	101 (15.7%)
Richer	160 (21.2%)	35 (17.6%)	493 (21.5%)	101 (20.7%)	568 (19.6%)	122 (19.5%)	678 (18.7%)	191 (29.7%)
Richest	296 (39.2%)	140 (70.4%)	567 (24.7%)	287 (58.7%)	636 (22.0%)	364 (58.1%)	375 (10.3%)	224 (34.8%)

^1^Year 2000 didn’t have any data on wealth index

In private health facilities, the proportion of childbirth increased from 1.4% to 14.5% during the study period. Urban women had a higher percentage of delivering in private health facilities than rural women (Fig. 1). Among the wealth index groups, although the percentage of the richest group using private facilities for childbirth significantly decreased from 70.4% in 2005 to 34.8% in 2021, they still frequented private facilities more than other groups (Table 2).

### Change of C-section rate over time

**Fig. 2:**
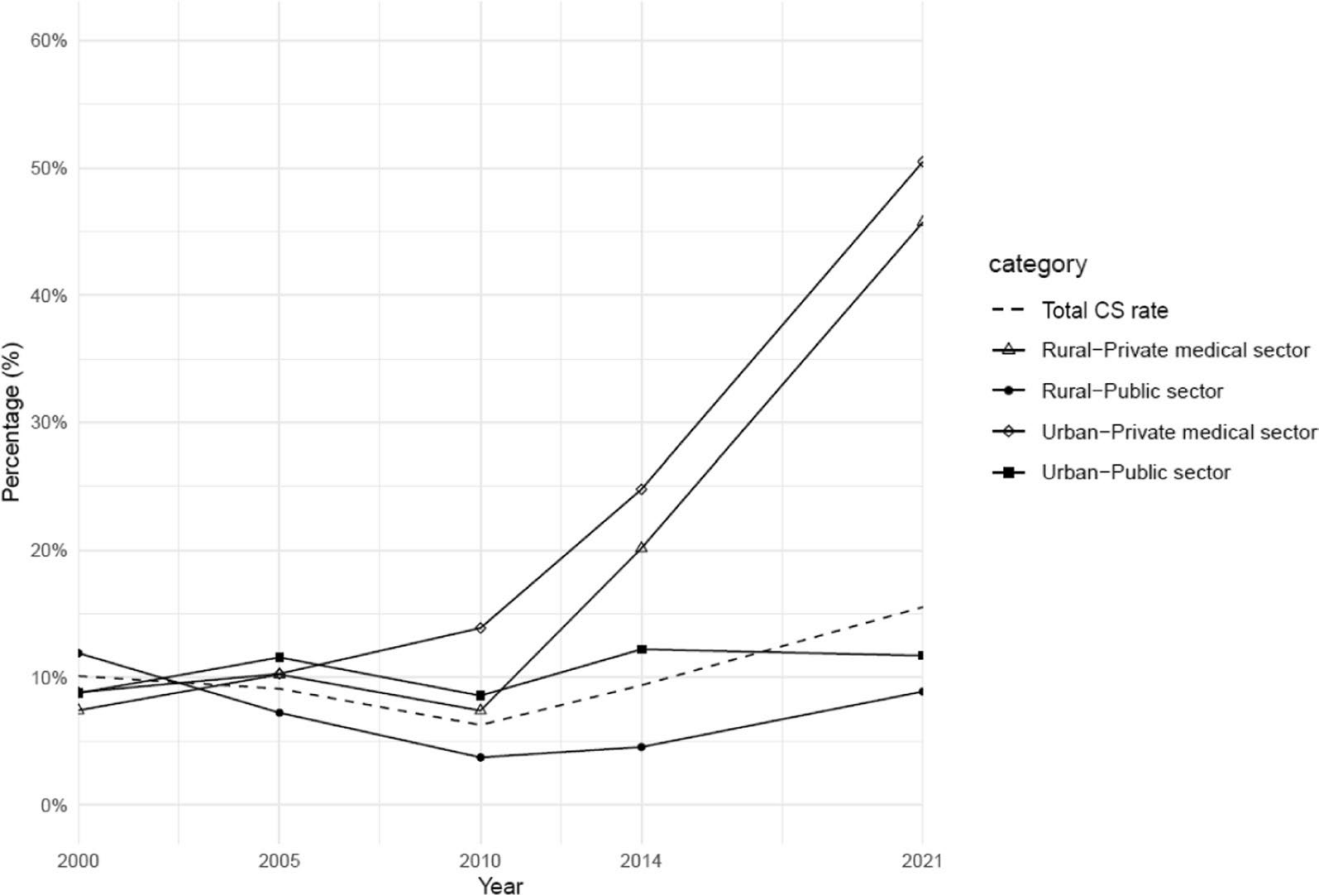
Changes of C-section rate by urban/rural, 2000–2021

The overall C-section rate in Cambodia initially declined from 10.1% in 2000 to 6.3% in 2010, then rose significantly to 15.5% by 2021 (Fig. 2). A similar trend was observed in public health facilities, where the C-section rate dropped from 10.6% in 2000 to 5.1% in 2010, before increasing to 9.7% by 2021 (Table 3). Since 2005, C-section rates in urban public health facilities consistently exceeded those in rural areas. During the period from 2005 to 2014, women from the richest group had the highest C-section rates in public facilities. By 2021, however, the distribution of C-section rates had become more evenly distributed across different wealth index groups (Table 4).

**Table 3:** C-section rate by socio-demographic characteristics, place of delivery and obstetrical history, 2000–2021

	Caesarian section						
	2000 (N = 38) n (%)	2005 (N = 88) n (%)		2010 (N = 172) n (%)	2014 (N = 328) n (%)	2021–22 (N = 664) n (%)	P-value
Socio-demographic characteristics							
*Residence*^1^							
Urban	15 (2.4%)	42 (4.5%)		99 (8.5%)	173 (15.3%)	287 (19.9%)	< 0.001
Rural	22 (0.6%)	43 (1.2%)		73 (2.2%)	152 (5.2%)	373 (12.5%)	
*Region*^**2**^							
Central Plain^3^	19 (1.5%)	33 (2.8%)		68 (5.3%)	132 (11.3%)	273 (21.2%)	< 0.001
Tonle Sap^4^	10 (0.6%)	31 (1.9%)		58 (3.5%)	96 (6.8%)	170 (12.8%)	
Coastal and Sea^5^	4 (0.8%)	10 (2.3%)		19 (4.4%)	42 (10.8%)	108 (17.9%)	
Plateau and Mountains^6^	4 (0.4%)	11 (0.9%)		27 (2.4%)	55 (5.2%)	109 (9.0%)	
*Maternal age*							
19 and less	4 (1.2%)	5 (1.4%)		10 (2.8%)	17 (4.6%)	22 (6.1%)	< 0.001
20–29	18 (0.9%)	48 (2.0%)		97 (3.4%)	180 (7.2%)	321 (13.7%)	
30+	16 (0.8%)	35 (2.0%)		65 (4.9%)	131 (10.9%)	321 (18.3%)	
*Religion*							
Buddhist	37 (0.9%)	84 (2.0%)		167 (3.9%)	317 (8.2%)	641 (15.0%)	< 0.001
Other^7^	1 (0.3%)	4 (1.2%)		5 (1.6%)	11 (4.7%)	23 (13.2%)	
*Education*							
No education	4 (0.2%)	12 (1.0%)		11 (1.2%)	18 (3.4%)	44 (7.9%)	< 0.001
Incomplete primary	13 (0.6%)	34 (1.5%)		52 (2.6%)	84 (5.5%)	143 (10.6%)	
Primary^8^	19 (2.7%)	30 (3.4%)		70 (5.0%)	148 (9.0%)	307 (15.6%)	
Secondary and higher^9^	2 (5.3%)	12 (14.8%)		39 (17.6%)	78 (21.3%)	170 (28.9%)	
*Health insurance type*							
No insurance	–	–		152 (4.0%)	287 (8.3%)	514 (14.7%)	1
Social^10^	–	–		13 (1.8%)	30 (5.6%)	116 (15.9%)	
Private	–	–		5 (6.8%)	11 (12.5%)	35 (15.8%)	
*Wealth*^**11**^							
Poorest	–	13 (1.0%)		11 (1.0%)	29 (3.1%)	92 (7.0%)	< 0.001
Poorer	–	9 (0.9%)		14 (1.6%)	18 (2.4%)	104 (12.1%)	
Middle	–	8 (1.0%)		14 (1.8%)	29 (4.5%)	115 (14.7%)	
Richer	–	10 (1.4%)		29 (3.6%)	62 (8.5%)	161 (18.2%)	
Richest	–	48 (7.1%)		104 (10.8%)	190 (18.5%)	192 (31.8%)	
*Partner’s education*^12^							
No education	4 (0.4%)	10 (1.3%)		11 (1.9%)	8 (2.1%)	24 (5.5%)	< 0.001
Incomplete primary	6 (0.3%)	16 (0.8%)		36 (2.2%)	62 (4.6%)	136 (11.0%)	
Primary	27 (1.8%)	33 (2.3%)		64 (3.7%)	140 (8.7%)	252 (14.2%)	
Secondary and higher	1 (3.3%)	27 (10.0%)		57 (10.6%)	117 (16.4%)	210 (28.0%)	
*Sex of household head*							
Male	28 (0.8%)	69 (1.8%)		140 (3.8%)	247 (7.9%)	448 (13.6%)	< 0.001
Female	10 (1.4%)	19 (2.8%)		32 (3.5%)	81 (8.7%)	216 (18.6%)	
*Decision-making on own health care*^13^							
Independently	-	-		74 (4.3%)	149 (9.3%)	256 (15.4%)	0.08
Jointly^14^	-	-		80 (3.5%)	143 (7.3%)	318 (14.5%)	
Not involve	-	-		13 (3.2%)	28 (8.2%)	60 (14.6%)	
*Decision-making on household*^15, 16^							
Independently	–	–		18 (6.1%)	23 (10.1%)	44 (18.0%)	0.2
Not entirely independent^17^	–	–		79 (3.6%)	192 (8.7%)	322 (14.3%)	
Jointly	–	–		66 (3.6%)	101 (7.3%)	247 (15.4%)	
Not involve	–	–		2 (3.6%)	3 (7.5%)	19 (12.7%)	
Place of delivery^18^							
*Public sector*^19^	***33 (10.6%)***	***67 (8.9%)***		***118 (5.1%)***	***190 (6.6%)***	***353 (9.7%)***	< 0.001
Public hospital	33 (12.8%)	62 (12.9%)		105 (11.7%)	181 (16.3%)	262 (17.9%)	
Health center/post	-	2 (0.8%)		12 (0.9%)	8 (0.4%)	88 (4.1%)	
*Private medical sector*^20^	***5 (8.1%)***	***21 (10.6%)***		***54 (11.0%)***	***138 (22.0%)***	***309 (48.1%)***	
Private hospital	3 (13.0%)	5 (14.7%)		17 (17.2%)	56 (28.1%)	90 (47.9%)	
Private clinic	2 (5.6%)	14 (10.5%)		34 (9.5%)	78 (20.8%)	215 (48.0%)	
Maternal and child related							
*Number of antenatal visits*^21^							
0	6 (0.2%)	5 (0.4%)		3 (0.6%)	1 (0.5%)	12 (14.0%)	< 0.001
1–3	21 (1.4%)	35 (1.7%)		28 (2.1%)	47 (5.6%)	62 (8.7%)	
4–7	9 (2.9%)	41 (3.9%)		104 (4.6%)	194 (8.3%)	385 (14.4%)	
8+	2 (9.5%)	6 (6.9%)		37 (8.6%)	82 (11.7%)	203 (20.9%)	
*Parity*							
1	10 (1.3%)	36 (3.2%)		75 (5.0%)	155 (9.7%)	247 (17.3%)	< 0.001
2	12 (1.5%)	25 (2.4%)		53 (4.3%)	100 (8.1%)	238 (14.7%)	
3+	16 (0.6%)	27 (1.2%)		44 (2.4%)	73 (5.9%)	179 (12.7%)	
*Size of child at birth*^22^							
Average	12 (0.5%)	28 (1.4%)		67 (3.3%)	161 (7.4%)	289 (13.5%)	< 0.001
Larger than average	19 (1.5%)	50 (2.8%)		86 (4.6%)	133 (9.3%)	331 (16.9%)	
Smaller than average	6 (0.9%)	9 (1.3%)		18 (3.6%)	33 (7.5%)	44 (13.3%)	
*Twins or multiple birth*							
Yes	3 (5.4%)	2 (4.7%)		4 (10.3%)	13 (34.2%)	9 (36.0%)	< 0.001
No	35 (0.8%)	86 (1.9%)		168 (3.7%)	315 (7.8%)	655 (14.8%)	
*Sex of child*							
Male	24 (1.1%)	52 (2.3%)		89 (3.8%)	157 (7.6%)	329 (14.4%)	0.7
Female	14 (0.6%)	36 (1.6%)		83 (3.7%)	171 (8.5%)	335 (15.5%)	

^1^One women in 2000, three in 2005, three in 2014 and four in 2021 had missing value

^2^One women in 2000, three in 2005, three in 2014 and four in 2021 had missing value

^3^Central Plain includes Kampong Cham, Tbong Khmum, Kandal, Phnom Penh, Prey Veng, Svay Rieng, and Takeo

^4^Tonle Sap includes Banteay Meanchey, Battambang, Kampong Chhnang, Kampong Thom, Pursat, Siem Reap, Otdar Meanchey, and Pailin

^5^Coastal and Sea includes Kampot, Koh Kong, Preah Sihanouk, and Kep

^6^Plateau and Mountains includes Kampong Speu, Kratie, Mondul Kiri, Preah Vihear, Ratanak Kiri, and Stung Treng

^7^Other religion includes Moslem, Christian, other religion and no religion

8Primary includes complete primary and incomplete secondary

^9^Secondary and higher includes complete secondary and higher

^10^Social health insurance includes National Social Security Fund and Health Equity Fund

^11^Year 2000 didn’t have any data on wealth index

^12^Two women’s partner in 2005, four in 2010, one in 2014 and 42 in 2021 had missing value

^13^Five women in 2010, eight in 2014 and 30 in 2021 had missing value

^14^Jointly includes respondent and husband/partner and respondent and other person

^15^Decision-making on household includes making large household purchases, visits to family or relatives and what to do with husband earns

^16^Seven women in 2010, nine in 2014 and 32 in 2021 had missing value

^17^Women can independently make decision on at least one of household affairs

^18^Two women in 2021 had missing value

^19^Three women in 2005, one in 2010 and three in 2021 had missing value

^20^Two women in 2005, three in 2010, four in 2014 and four in 2021 had missing value

^21^One woman in 2005, four in 2014 and two in 2021 had missing value

^22^One woman in 2000, one in 2005, one in 2010 and one in 2014 had missing value

**Table 4:** C-section rate in public/private facilities by wealth index, 2005–2021

	C-section rate							
	2005		2010		2014		2021–22	
	Public (N = 67) n (%)	Private (N = 21) n (%)	Public (N = 118) n (%)	Private (N = 54) n (%)	Public (N = 190) n (%)	Private (N = 138) n (%)	Public (N = 353) n (%)	Private (N = 309) n (%)
*Wealth*^1^								
Poorest	13 (19.4%)	**–**	11 (9.3%)	**–**	26 (13.7%)	3 (2.2%)	74 (21.0%)	18 (5.8%)
Poorer	8 (11.9%)	1 (4.8%)	13 (11.0%)	1 (1.9%)	14 (7.4%)	4 (2.9%)	59 (16.7%)	45 (14.6%)
Middle	8 (11.9%)	**–**	11 (9.3%)	3 (5.6%)	14 (7.4%)	15 (10.9%)	77 (21.8%)	38 (12.3%)
Richer	8 (11.9%)	2 (9.5%)	23 (19.5%)	6 (11.1%)	40 (21.1%)	22 (15.9%)	70 (19.8%)	89 (28.8%)
Richest	30 (44.8%)	18 (85.7%)	60 (50.8%)	44 (81.5%)	96 (50.5%)	94 (68.1%)	73 (20.7%)	119 (38.5%)

^1^Year 2000 didn’t have any data on wealth index

From 2000 to 2021, private health facilities experienced a pronounced increase in C-section rates, rising nearly sixfold to 48.1% in 2021 (Table 3). In both urban and rural settings, significant increases were observed in private facilities. Urban C-section rates rose from 8.8% in 2000 to 50.5% in 2021, while rural rates increased from 7.4% to 45.7% over the same period (Fig. 2). In 2021, the richest and richer groups accounted for most C-section deliveries in private facilities, constituting 38.5% and 28.8%, respectively (Table 4).

Over the entire study period, all socio-demographic groups experienced increases in C-section rates (Table 3). The most pronounced increases were observed among women over 30, had higher education levels, with female household head and had at least eight antenatal visits. Notably, the proportion of C-sections among women with only one child increased significantly from 1.3% in 2000 to 17.3% in 2021 (Table 3).

### Determinants of C-section

After adjusting for explanatory variables, the C-section usage was significantly higher in 2021 compared to 2010 (Adjusted OR 3.22, 95% CI [2.72, 4.07]). Women who delivered in private health facilities had a 3.74 (95% CI [3.21, 4.35]) increased odds of undergoing a C-section compared to those in public health facilities. After adjusting for year and place of delivery, women over 20 years old, living in Central Plain, from richer or richest households, had secondary and higher education level or with female household head were more likely to undergo a C-section than other women. Additionally, women with only one child, had twins or multiple births or the size of child at birth was larger than average were more likely to have a C-section than other women. However, after adjusting for all explanatory variables, no statistically significant association was found between having at least eight antenatal visits and C-section use (Table 5).

**Table 5:** Factors associated with use of caesarean section in Cambodia, 2010 –2021^1^

Variables	Unadjusted OR (95%CI)	Adjusted^2^OR (95%CI)	p-value
*Year*			
2010	Ref	Ref	
2014	2.18 (1.80, 2.65)	1.57 (1.28, 1.93)	< 0.001
2021	4.36 (3.66, 5.22)	3.32 (2.72, 4.07)	< 0.001
Socio-demographic characteristics			
*Maternal age*			
19 and less	Ref	Ref	
20–29	1.77 (1.32, 2.45)	1.74 (1.26, 2.44)	< 0.001
30+	2.90 (2.15, 4.00)	4.10 (2.90, 5.91)	< 0.001
*Region*			
Plateau and Mountains^3^	Ref	Ref	
Central Plain^4^	2.52 (2.11, 3.02)	1.36 (1.12, 1.67)	0.002
Coastal and Sea^5^	2.39 (1.91, 2.99)	1.27 (0.99, 1.62)	0.06
Tonle Sap^6^	1.33 (1.10, 1.61)	1.20 (0.98, 1.48)	0.08
*Wealth*			
Poorest	Ref	Ref	
Poorer	1.45 (1.12, 1.87)	1.06 (0.81, 1.39)	0.65
Middle	1.87 (1.46, 2.39)	1.14 (0.87, 1.49)	0.34
Richer	2.89 (2.31, 3.63)	1.43 (1.12, 1.85)	0.005
Richest	5.84 (4.76, 7.20)	2.42 (1.88, 3.13)	< 0.001
*Education*			
No education	Ref	Ref	
Incomplete primary	1.45 (1.12, 1.91)	1.01 (0.76, 1.35)	0.94
Primary^7^	2.88 (2.25, 3.74)	1.17 (0.89, 1.56)	0.28
Secondary and higher^8^	8.05 (6.17, 10.63)	1.75 (1.27, 2.42)	< 0.001
*Sex of household head*			
Male	Ref	Ref	
Female	1.35 (1.17, 1.55)	1.20 (1.03, 1.40)	0.02
Child birth			
*Place of delivery*			
Public sector	Ref	Ref	
Private sector	5.04 (4.40, 5.76)	3.74 (3.21, 4.35)	< 0.001
Maternal and child related			
*Parity*			
1	Ref	Ref	
2	0.90 (0.78, 1.04)	0.65 (0.55, 0.77)	< 0.001
3+	0.60 (0.51, 0.70)	0.42 (0.34, 0.52)	< 0.001
*Size of child at birth*			
Average	Ref	Ref	
Larger than average	1.33 (1.17, 1.51)	1.18 (1.02, 1.36)	0.02
Smaller than average	0.86 (0.67, 1.09)	1.04 (0.79, 1.35)	0.77
*Twins or multiple birth*			
No	Ref	Ref	
Yes	3.66 (2.27, 5.72)	7.01 (4.02, 11.94)	< 0.001

^1^The *p* value of Hosmer–Lemeshow Test is 0.1113, indicating a good fit of the model to the data

^2^Adjusting for all explanatory variables

^3^Plateau and Mountains includes Kampong Speu, Kratie, Mondul Kiri, Preah Vihear, Ratanak Kiri, and Stung Treng

^4^Central Plain includes Kampong Cham, Tbong Khmum, Kandal, Phnom Penh, Prey Veng, Svay Rieng, and Takeo

^5^Coastal and Sea includes Kampot, Koh Kong, Preah Sihanouk, and Kep

^6^Tonle Sap includes Banteay Meanchey, Battambang, Kampong Chhnang, Kampong Thom, Pursat, Siem Reap, Otdar Meanchey, and Pailin

^7^Primary includes complete primary and incomplete secondary

^8^Secondary and higher includes complete secondary and higher

## Discussion

### Main findings

In Cambodia, facility-based delivery increased significantly between 2000 and 2021. Urban women and those from the richest households had a higher percentage of delivering in private health facilities, whereas rural women and those from the poorest households more commonly utilized public health facilities. From 2010 to 2021, there was a marked increase in C-section rates, primarily in private health facilities and among women from wealthier economic groups. Notably, women from the richest group recorded the highest C-section rates in both public and private facilities. In 2021, public health facilities showed no significant differences in C-section utilization across different wealth index groups. Moreover, there was a rising trend in C-section rates among women having only one child over the years.

### Interpretation

Consistent with trends in other low- and middle-income countries [28, 29], Cambodia experienced a substantial increase in facility-based deliveries from 2000 to 2021, supported by policies that addressed both supply and demand barriers. On the supply side, key initiatives included training and integrating traditional birth attendants into the health system and providing financial incentives to encourage skilled birth attendants to facilitate deliveries in public facilities [30–32]. Demand-site efforts have focused on improving access to health services through various financial support schemes [31]. Women from households with an ID Poor Card can access essential maternity care (including C-sections) in public facilities for free. These services are funded by the government Health Equity Fund [31, 33]. Moreover, community-based health insurance with an affordable premium targets near-poor populations, covering user fees at contracted public providers [34]. In addition, women with formal employment are covered by the national social health insurance, known as the National Social Security Fund, although the proportion of these women is low. The National Social Security Fund provides similar maternity care benefits at designated partner facilities, which include both public and private health sectors [35, 36].

Between 2014 and 2021, rural areas showed higher use of public facilities, while private facilities were more commonly utilized in urban areas. To address the urban–rural disparity in health service coverage, the Cambodian government has established new primary public health centers in rural areas, taking into account local population size and geographic accessibility [37]. This may explain the significant improvement in public facility-based deliveries in rural areas, attributed to lower costs and improved accessibility [33, 37, 38]. In recent years, private healthcare facilities have expanded rapidly in Cambodia, primarily concentrated in urban areas [37]. Urban women who are willing and able to pay for maternity care are more likely to opt for private health facilities, which are often perceived as offering higher-quality care [37].

With the achievement of near-universal facility-based deliveries in Cambodia, there has also been a rapid increase in C-section rates. Schantz’s study [39] suggests that some Cambodian women may request C-section due to the preference for selecting an auspicious birth date, fear of vaginal birth, and/or fear of labor pains. These factors are common reasons for C-sections performed without medical indications in many low- and middle-income countries [40–42]. In public health facilities, regulations surrounding the performance of C-sections dictate that maternity care providers must counsel and often try to dissuade pregnant women from electing for a C-section when it is not medically necessary. This guidance may help to control the C-section rate in public health facilities, which stood at 9.7% in 2021, showing no significant differences across women’s socio-economic statuses.

Although the national strategies emphasize the importance of collaboration between the public and private sectors, there is often a lack of mechanisms for coordination, regulating, and monitoring the performance and quality of care in private health sector [33, 43]. In 2021, the C-section rate in private health facilities surged to 48.1%, raising concerns about potential overuse. Profit-driven practices are a potential factor contributing to the high C-section rates, which has been observed in private health facilities across Latin America and other low and middle-income countries in Asia [44–47]. Schantz’s study [39] highlighted the cost of C-Section (278 USD) was almost five times the cost of vaginal births (60 USD) in a hybrid private–public university hospital in Cambodia. In the absence of strong regulatory oversight for private health sector, financial incentives may drive provider-induced demand or increase physicians' willingness to comply with maternal requests for C-Sections [44].

Dual practice, where healthcare providers work in both public and private sectors is common in many low- and middle-income countries [48, 49]. The evidence suggested that it could compromise the quality and availability of services in public facilities [48–50]. In Cambodia, well-trained public maternity care providers often serve as private practitioners to supplement their income [33, 43]. This practice may further lead to supply-induced demand for C-sections as elective C-sections can be more easily planned and managed when stringent regulation is lacking [39]. In addition, the excessive workload caused by dual practices has heightened concerns about the provision of respectful childbirth care, especially in the public health sector. A WHO-led study conducted across four low- and middle-income countries [51] reported that one-third of women, particularly those who are socially and economically vulnerable, experienced mistreatment during childbirth. This included physical and verbal abuse, discrimination, and a lack of supportive care, among other issues suggesting inequalities in women’s experience during childbirth.

This study also noted a significant rise in C-section rate among nulliparous women in Cambodia during the study period. A prior caesarean delivery could pose potential risks in subsequent pregnancies, leading to greater medical care needs and higher healthcare system costs [39, 52]. In Cambodia, where most women have multiple births, this trend indicates a future increase in demand for intensive maternity care and repeated C-section procedures, posing a challenge that the healthcare system must prepare for [39]. Furthermore, well-educated women and those from household with female head were more likely to choose C-sections. A significant factor driving the high rates of C-sections linked to women's empowerment is their explicit request for these procedures. This preference is influenced by increased education, economic stability, and autonomy, which shape their choices regarding the mode of childbirth [52], particularly elective C-sections, which are often perceived as less painful or more convenient methods of delivery [53].

Based on findings from this study, Cambodia provides valuable insights for other low and middle-income countries, especially those with mixed public–private health systems facing similar challenges. Strengthened public sector governance through standard setting and regulation has helped contain the rise in C-section rates within public facilities. However, weak oversight in private facilities has allowed C-sections to increase rapidly and often without medical indication. Furthermore, widespread dual practice may adversely impact women’s childbirth experiences and diminish the quality of care in the public health sector. Hence, further studies should be conducted to explore mechanisms to strengthen health system governance with a focus on improving public and private sector coordination through performance-based incentives and enhancing monitoring and accountability of private health facilities to reduce unnecessary C-sections and ensure equitable access to quality maternity care.

### Strengths and limitations

This study investigated the changes in place of delivery and C-section rates over the past two decades in Cambodia, utilizing a nationally representative sample. The data quality was widely acknowledged as reliable [19–23]. However, this study also had some limitations. Recall bias is a potential concern, as women might not accurately remember past events. However, the significant nature of childbirth reduces the likelihood of serious recall issues, particularly for the most recent live birth. The size of the child at birth was subjectively reported by the respondents, which may differ from medical definitions. The surveys did not distinguish whether C-sections were elective. Furthermore, without detailed pregnancy histories such as previous C-sections or specific information during the pregnancy, it remains challenging to ascertain whether a C-section was performed for medical reasons.

## Conclusions

C-section rate has increased significantly in Cambodia over the past decade, with private health facilities exhibiting particularly alarming rates. Women with higher socio-economic status are more likely to undergo C-sections. It is essential to reinforce health system governance within the private sector and promote public–private partnerships to guarantee equitable and effective childbirth care experience. In addition, community-based childbirth campaigns, alongside health education and promotion regarding the advantages and disadvantages of different modes of delivery, should further equip women and their family members to make informed decisions together.

## Data Availability

Data is available in a public, open access repository. All data used in this study are publicly available upon request at https://www.dhsprogram.com/data/Access-Instructions.cfm.
